# Obituary

**Published:** 1849-09

**Authors:** 


### ARTICLE II.

### OBITUARY.

DIED.—At Utica, N. Y., on the 9th of Sept., of dysentery, AMARIAH BRIGHAM, M. D., Superintendent of the N. Y. State Lunatic Asylum' and Editor of the American Journal of Insanity, aged 51 years. Dr. B. was highly distinguished as a sound philosopher and able writer.

On the 2d of Sept., of Cholera, after an illness of a few hours, JNO. P. HARRISON, M. D., Prof. in the Med. College of Ohio, and one of the Editors Western Lancet, universally known to the profession by his long connection with medical teaching, and his extensive productions as an author.

Of Cholera, at St. Louis, THOMAS BARBOUR, M. D., Prof. of Obs. &c., in the University of the State of Missouri, and formerly Editor of the Missouri Medical Journal.

Of Cholera, at his residence in Chicago, in August last, JOHN E. O'LEARY, M. D.

Of Cholera, on the 27th of July, at Naperville, Ill., ERASTUS G. HOUGH, M. D.

Both of these were young men of good standing and fair promise for usefulness. Although not old in the service, having graduated at Rush Medical College in 1848, they fell at their posts.

